# Serum Survivin Levels and Outcome of Chemotherapy in Patients with Malignant Mesothelioma

**DOI:** 10.1155/2015/316739

**Published:** 2015-09-16

**Authors:** Katja Goričar, Viljem Kovač, Alenka Franko, Metoda Dodič-Fikfak, Vita Dolžan

**Affiliations:** ^1^Pharmacogenetics Laboratory, Institute of Biochemistry, Faculty of Medicine, University of Ljubljana, SI-1000 Ljubljana, Slovenia; ^2^Institute of Oncology Ljubljana, SI-1000 Ljubljana, Slovenia; ^3^Clinical Institute of Occupational Medicine, University Medical Center Ljubljana, SI-1000 Ljubljana, Slovenia

## Abstract

*Background.* Survivin is an inhibitor of apoptosis protein involved in the regulation of cell proliferation that could be used as a marker for cancer diagnosis or prognosis. Our aim was to evaluate whether serum survivin levels influence the outcome of cisplatin-based chemotherapy in patients with malignant mesothelioma (MM). *Methods.* Serum survivin levels were determined using human survivin enzyme-linked immunosorbent assay in 78 MM patients before chemotherapy, after chemotherapy, and at disease progression. The influence on tumor response and survival was evaluated using nonparametric tests and Cox regression. *Results.* A median serum survivin level at diagnosis was 4.1 (0–217.5) pg/mL. Patients with a progressive disease had significantly higher survivin levels before chemotherapy (*p* = 0.041). A median serum survivin level after chemotherapy was 73.1 (0–346.2) pg/mL. If survivin levels increased after chemotherapy, patients had, conversely, better response (*p* = 0.001, OR = 5.40, 95% CI = 1.98–14.72). Unexpectedly, patients with increased survivin levels after chemotherapy also had longer progression-free (*p* < 0.001, HR = 0.33, 95% CI = 0.20–0.57) and overall survival (*p* = 0.001, HR = 0.29, 95% CI = 0.14–0.58). *Conclusions.* These results suggest that serum survivin levels before and during chemotherapy could serve as a biomarker predicting MM treatment response.

## 1. Introduction

Survivin is a key member of the inhibitor of the apoptosis protein (IAP) family, encoded by the* BIRC5* (baculoviral inhibitor of apoptosis repeat containing 5) gene. Survivin is involved in the regulation of both apoptosis and cell division [1]. IAPs bind and inhibit caspases, reducing their activity and leading to suppression of programmed cell death [2]. The antiapoptotic role of survivin is also exerted through binding and stabilization of X-linked IAP (XIAP) [1, 3].

Survivin is usually not expressed in normal differentiated tissues but is highly expressed in several cancers [1]. The regulation of survivin expression is complex and includes alternative splicing, variability in transcription, or protein degradation and may vary during the cell cycle [1, 4]. Altered survivin expression in cancer can also be associated with the amplification of* BIRC5* locus, different methylation pattern, or differences in promoter activity [1]. Promitotic and antiapoptotic activity of survivin is also linked to its localization in the nucleus or the cytoplasm [5].

Survivin expression allows tumor cells to overcome apoptotic checkpoints and may play an important role in cancer progression [1, 6]. Survivin has been proposed as a marker not only for diagnosis, but also for prognosis in various cancers [7]. Increased survivin expression is associated with an altered disease outcome or survival in various cancers, but the results differ among studies and cancer types [8–14]. As several antitumor agents function through apoptosis activation, survivin expression may contribute to the resistance to anticancer agents [1] and could thus help to predict response to chemotherapy [8]. Studies have already shown that inhibition of survivin can sensitize tumor cells to different chemotherapeutic agents including cisplatin [15].

Cisplatin is used in chemotherapy of several cancer types, including malignant mesothelioma (MM). MM is a rare and aggressive malignancy associated with asbestos exposure that has an unfavorable prognosis and short survival. The use of cisplatin-based chemotherapy considerably improved the survival of MM patients [16, 17] and combinations with pemetrexed or gemcitabine had comparable outcomes [16, 18–21]. However, the median survival of MM patients is still short and usually fewer than half of the patients reach complete or partial response after chemotherapy [16, 22].

Previous studies in MM evaluated survivin expression in tissue samples or cell lines [23–28]. These studies confirmed both nuclear and cytoplasmic expression of survivin in MM tumors [23, 25–28], but the results regarding the association with the disease outcome were not concordant. Recent studies suggested that even though survivin expression was not associated with overall survival in MM [23], higher survivin expression in MM tumors was observed in patients with a better response to tumor-directed treatment [24].

Due to an increasing incidence of MM and its poor prognosis, new prognostic and predictive biomarkers, preferably noninvasive, are needed, as previously described biomarkers, such as mesothelin and fibulin-3, have only a limited prognostic ability [29, 30]. As studies in other cancer types already showed that survivin can also be measured in the serum [31–35], our aim was to evaluate whether serum survivin levels influence the outcome of cisplatin-based chemotherapy in patients with malignant mesothelioma.

## 2. Patients and Methods

### 2.1. Patients

This panel study included patients with histologically proven MM treated with cisplatin-based chemotherapy at the Institute of Oncology, Ljubljana, Slovenia, between 1 January 2007 and December 2013. Serum samples were collected before the start of the first day of chemotherapy, after the last cycle of chemotherapy, and at disease progression. Only two samples were collected from patients who did not experience disease progression within the observation period or from patients with disease progression as the immediate outcome of chemotherapy.

Demographic, clinical, and treatment data were obtained from the medical records. Smoking status and exposure to asbestos were obtained during the clinical interview. Regarding exposure to asbestos, patients were classified into the following groups: occupational, environmental, and occasional asbestos exposure or no known asbestos exposure. Informed consent was obtained from all patients. The study was approved by the Slovenian Ethics Committee for Research in Medicine and was carried out according to the Declaration of Helsinki.

### 2.2. Response and Survival Assessment

The primary outcome of interest was tumor response, evaluated using the modified Response Evaluation Criteria in Solid Tumors (RECIST) [36]. A complete or partial response was considered as a good response, while stable or progressive disease was considered as a poor response. When evaluating the disease control rate, patients with a complete or partial response or stable disease were compared to patients with progressive disease. In the survival analysis, progression-free survival (PFS) and overall survival (OS) were also assessed. PFS was defined as the time from the first day of chemotherapy to the day of documented disease progression, and overall survival (OS) time was defined as the time from the first day of chemotherapy to death from any cause. Patients without progression or death at the time of the analysis were censored at the date of the last follow-up.

### 2.3. Serum Survivin Level Measurement

Serum samples were prepared immediately after blood sampling, aliquoted, and stored at −20°C until survivin levels were measured. Serum survivin levels were determined using a human survivin enzyme-linked immunosorbent assay (ELISA) kit according to the manufacturer's instructions (Boster Biological Technology, Fremont, CA, USA) blinded regarding the study endpoints.

### 2.4. Statistical Analyses

To describe central tendency and variability, median and interquartile ranges were used. Nonparametric Kruskal-Wallis and Mann-Whitney tests were used to compare differences in serum survivin concentrations. Pairwise comparisons with* post hoc* Bonferroni corrections were used to determine which of the treatment outcome groups significantly differed between each other with Kruskal-Wallis test. The Cox proportional hazards model was used to determine the hazard ratios (HR) with the 95% confidence intervals (CIs) in the survival analysis. The Kaplan-Meier analysis was used to calculate the median survival times. A receiver operating characteristic (ROC) curve was used to analyze the predictive value of survivin and the area under the curve (AUC) was determined. The predicted and observed responses were compared to calculate the specificity, sensitivity, positive predictive value (PPV), and negative predictive value (NPV). The value with the highest sum of sensitivity and specificity was selected as the cutoff. Logistic regression was used to calculate odds ratios and 95% CIs for determining the influence of categorical variables on the response rate. All statistical analyses were performed using IBM SPSS Statistics, version 19.0 (IBM Corporation, Armonk, NY, USA). All statistical tests were two-sided and the level of significance was set at 0.05.

## 3. Results

In total, 78 MM patients were included in the study. For all patients, pretreatment and posttreatment samples were available. Serum samples at disease progression were available for 61 (78.2%) patients. Median follow-up time was 25.2 months. An additional ten patients experienced disease progression by the time of the analysis; however, samples were not available for the inclusion in the study. Progression therefore occurred in a total of 71 (91.0%) patients, while 42 (53.8%) patients died by the time of the analysis. The patients' characteristics are presented in Table 1.

Serum survivin levels differed considerably among patients. A median serum survivin level was 4.1 pg/mL (0–217.5 pg/mL) at diagnosis, 73.1 pg/mL (0–346.2 pg/mL) after chemotherapy, and 28.3 pg/mL (0–366.9 pg/mL) at disease progression. In 35 (44.9%) patients, serum survivin levels increased after cisplatin-based chemotherapy. The median change of survivin levels after chemotherapy was 0 pg/mL (−4.1 to 113.7 pg/mL). At disease progression, serum survivin levels were also higher compared to baseline in 22 (36.1%) out of 61 patients; the median change was 0 pg/mL (0–81.6 pg/mL).

We also compared serum survivin levels at diagnosis among patients with different clinical characteristics. MM patients without known asbestos exposure had significantly higher serum survivin levels at diagnosis (Mann-Whitney *U* = 270.0, *p* = 0.006): 303.8 pg/mL (0–2432.7 pg/mL) compared to 0 pg/mL (0–87.4 pg/mL) in patients exposed to asbestos. Further analyses were therefore performed separately also for the cohort of patients exposed to asbestos.

### 3.1. Treatment Outcome

Survivin levels before chemotherapy and a change in survivin levels after chemotherapy are presented in Table 2. Higher serum survivin levels before chemotherapy were observed in patients with a worse disease outcome (Kruskal-Wallis test statistic = 7.951, *p* = 0.047, Table 2). Patients who experienced disease progression during chemotherapy had higher survivin levels compared to patients with disease control (Mann-Whitney *U* = 354.0, *p* = 0.041) and this difference was more pronounced if only patients exposed to asbestos were evaluated (Mann-Whitney *U* = 190.0, *p* = 0.021).

Patients with increased serum survivin levels had a better response to platinum-based chemotherapy, while serum survivin levels decreased in patients with progressive disease (Kruskal-Wallis test statistic = 8.892, *p* = 0.031, Table 2). This observation was even more significant if only patients exposed to asbestos were evaluated (Kruskal-Wallis test statistic = 14.365, *p* = 0.002, Table 2). Differences between a progressive disease and a complete response and between a progressive disease and a partial response remained significant after Bonferroni correction (Kruskal-Wallis test statistic = 39.500 and 31.579, adjusted *p* = 0.010 and 0.007, resp.).

A median change in the serum survivin level was 61.6 pg/mL (0–215.4 pg/mL) in patients with a good response and 0 pg/mL (−34.4–54.3 pg/mL) in patients with a poor response. When comparing patients with a good and poor response, significant differences in the change in the serum survivin level after chemotherapy were observed in the whole patient cohort and among patients exposed to asbestos (Mann-Whitney *U* = 908.0, *p* = 0.024, and Mann-Whitney *U* = 643.0, *p* = 0.004, resp.). Similarly, the change in survivin levels differed significantly between patients with progressive disease and patients with disease control (Mann-Whitney *U* = 123.0, *p* = 0.028, for all patients, and Mann-Whitney *U* = 18.0, *p* = 0.004, for patients exposed to asbestos).

**Figure 1:**
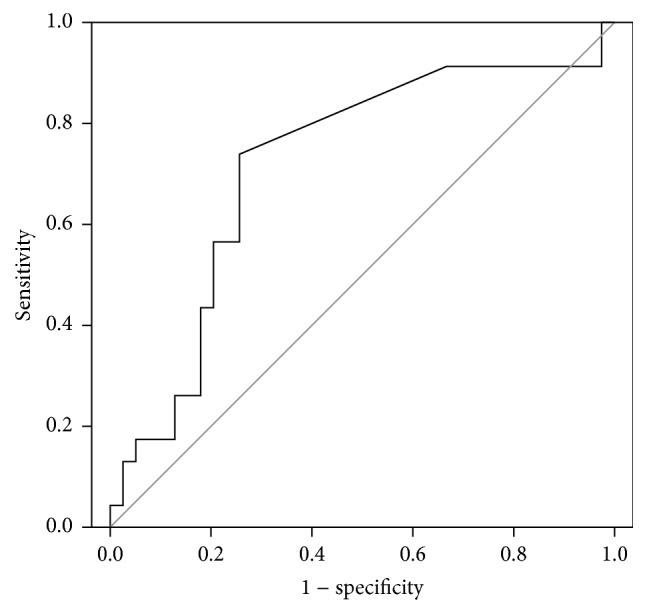
Receiver operating characteristic curve for the change in survivin levels predicting the response rate in malignant mesothelioma patients.

To determine the cutoff value for the change of the serum survivin level after chemotherapy, we calculated the specificity and sensitivity of predicting the response rate. The cutoff value was 0 and patients with an increase in survivin levels had 5.40 times greater odds for a good response than patients with a decrease in survivin levels (*p* = 0.001, OR = 5.40, 95% CI = 1.98–14.72). When all patients were assessed, this cutoff had a specificity of 0.708 and sensitivity of 0.690, while PPV was 0.588 and NPV was 0.791 (Table 3). Among asbestos exposed patients, the AUC for the change in survivin levels predicting the response rate was 0.717 (95% CI = 0.580–0.854) (*p* = 0.005, Figure 1). At the cutoff value of 0, the specificity was 0.744 and sensitivity was 0.739, while PPV was 0.630 and NPV was 0.829 (Table 3). If serum survivin levels increased after chemotherapy, patients had 8.22 times greater odds for a good response (*p* < 0.001, OR = 8.22, 95% CI = 2.54–26.63).

### 3.2. Survival Analysis

A higher serum survivin level before chemotherapy did not have a substantial effect on PFS among all patients (*p* = 0.202, HR = 1.01, 95% CI = 0.99–1.04, for a survivin increase of 100 pg/mL) or patients exposed to asbestos (*p* = 0.046, HR = 1.03, 95% CI = 1.00–1.05, for a survivin increase of 100 pg/mL). Additionally, there was no association with OS (*p* = 0.444 for all patients and *p* = 0.194 for patients exposed to asbestos).

**Figure 2:**
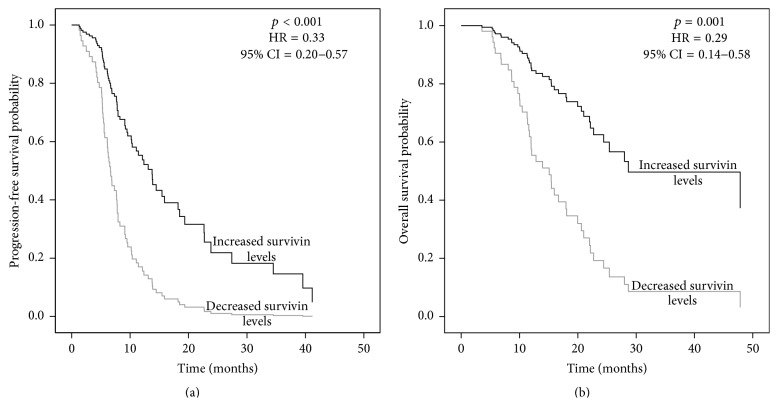
Change in serum survivin levels after chemotherapy is associated with (a) progression-free survival and (b) overall survival of malignant mesothelioma patients.

We compared patients with increased survivin levels after chemotherapy compared to pretreatment levels to patients with unchanged or decreased levels in the survival analysis. Patients with increased survivin levels after chemotherapy had significantly longer PFS both in the whole cohort of patients and in the subgroup exposed to asbestos (*p* < 0.001, HR = 0.33, 95% CI = 0.20–0.57, Figure 2(a), and *p* = 0.002, HR = 0.40, 95% CI = 0.22–0.71, resp.). Similarly, increased survivin levels were also associated with longer OS in the whole cohort (*p* = 0.001, HR = 0.29, 95% CI = 0.14–0.58, Figure 2(b)) and among patients with known asbestos exposure (*p* = 0.008, HR = 0.35, 95% CI = 0.16–0.77). In the whole cohort of patients, those with decreased survivin levels had median PFS of 6.7 (5.2–9.6) months and median OS of 15.1 (10.0–22.0) months, compared to 13.1 (2.8–23.8) months and 47.8 (16.3–47.8) months in patients with increased survivin after chemotherapy. In the asbestos exposed patients, the ones with decreased survivin levels had median PFS of 6.7 (5.2–9.6) months and median OS of 15.1 (11.0–21.0) months, compared to 10.4 (6.5–23.8) months and 47.8 (18.0–47.8) months in patients with increased survivin after chemotherapy.

Regarding the change in survivin levels at disease progression, we only evaluated their influence on OS: if survivin levels increased compared to baseline, patients had significantly longer overall survival (*p* = 0.001, HR = 0.84, 95% CI = 0.76–0.93, for survivin increase of 100 pg/mL); however, only a trend towards longer OS was seen in the subgroup exposed to asbestos (*p* = 0.092, HR = 0.88, 95% CI = 0.67–1.02, for a survivin increase of 100 pg/mL).

## 4. Discussion

In the present study, we evaluated serum survivin levels in MM patients before and after cisplatin-based chemotherapy. Patients with higher survivin levels before chemotherapy tended to have a worse treatment outcome; if the levels increased after chemotherapy, the results show the opposite direction: patients had a better treatment outcome and longer PFS and OS.

Survivin is an interesting potential biomarker in cancer due to its role in the regulation of apoptosis and cell division [1] as inhibition of apoptosis could contribute to cancer progression, but it could also affect cancer treatment. Several studies therefore investigated whether it could be used as a prognostic or predictive biomarker in MM. The first studies in MM showed that survivin mRNA levels were significantly higher in pleural MM tissue and inflammatory tissue [25], but the association with the disease outcome was not the same in all studies. Survivin positive tumors were associated with shorter survival in one of the studies [26], but other studies showed no association with the disease outcome [23, 27]. Conversely, patients treated with chemotherapy alone in combination with radiotherapy or surgery had significantly higher tumor survivin expression than patients that received the best supportive care or palliative treatment or were not treated at all [24]. These observations are in accordance with the data showing that the treatment with cisplatin leads to increased survivin expression in MM cell lines [37]. Additionally, even though higher nuclear survivin expression in tumor samples or* BIRC5* polymorphisms was not related to OS in all patients regardless of the treatment [23], higher survivin expression was related to a better treatment outcome in patients treated with chemotherapy [24]. The latter is in accordance with our results, where patients with increased survivin expression after chemotherapy had a better response to chemotherapy and longer survival.

Previous studies investigating serum survivin levels in other malignancies focused mostly on levels at diagnosis and comparison to healthy individuals [31–35]. Consistently, survivin levels were higher in patients compared to controls, even though the difference was not always statistically significant. In ovarian cancer, gallbladder cancer, and pancreatic ductal adenocarcinoma, higher pretreatment levels of survivin were also associated with shorter survival [33–35]. In accordance with these results, patients with higher serum survivin levels at diagnosis also tended to have a worse outcome in our study. On the other hand, only two studies compared survivin levels before and after treatment: no difference was observed in breast cancer or non-small cell lung cancer, but the association between a change in expression and a treatment outcome was not examined [32, 38].

Most of the other studies focused on survivin expression in the tumor at diagnosis, where it was usually regarded as a marker of a poor response. For example, survivin negative tumors were associated with better survival rates in several cancer types, including bladder cancer, colorectal cancer, medulloblastoma, and glioma [8, 11–13]. But the results of several studies suggest that survivin has a more complex role in cancer, as higher survivin expression was also often associated with a favorable outcome [14, 39–43]. In most of these studies, the nuclear and cytoplasmic localization of survivin was assessed separately as it might influence its antiapoptotic activity [5]. Nuclear expression was generally associated with longer survival [40–42]. In studies focused on specific cancer treatment, higher survivin expression in tumor was associated with a better response to radiotherapy and longer survival in head and neck or oral squamous cell carcinoma [14, 39]. In cell lines, survivin silencing also led to decreased sensitivity to radiation [14]. In non-small cell lung cancer, higher nuclear survivin expression was also associated with longer survival after chemotherapy [43], which is also in agreement with our results suggesting that higher survivin levels after chemotherapy may be associated with a better treatment response. On the other hand, the opposite effect was observed in other studies where survivin silencing increased the sensitivity to chemotherapy or radiation [44], further suggesting that survivin might have additional molecular functions. The biological basis for the role of survivin in the treatment response is still not well established. It was proposed that high survivin expression might be associated with higher susceptibility to DNA damage induced by cancer treatment [39]. Recent studies show that survivin interacts with components of DNA double-strand break repair and might regulate DNA repair [44]. Moreover, induction of DNA damage leads to increased survivin expression [45]. Different functions of survivin could help to explain the results of a study on breast cancer, where high survivin levels were associated with a poor response to endocrine treatment, but a good response to chemotherapy [46].

Differences in survivin localization, as well as cancer type and cancer treatment, might therefore affect the role of survivin expression in cancer diagnosis and prognosis. Consequently, careful selection of tumor or serum biomarkers, determination of appropriate cutoff values, and validation of the results are very important before the implementation in the clinical practice. In our pilot study, we focused on serum survivin levels as they are easily determined using a noninvasive approach and amenable to follow-up. ELISA was selected as an appropriate method for serum survivin detection. As a commercially available kit was used, we did not validate the results with other methods, which represents a limitation of our study. Previous studies show that different commercially available kits may have different sensitivity; however, in their setting, no difference was observed between cancer patients and controls in regard to the levels of serum survivin detected by two different ELISA kits [47]. Before potential implementation in the clinical practice, further studies should therefore validate which commercially available assays are the most sensitive and reliable in the detection of a serum pool of survivin in MM patients and have higher predictive values in serum samples.

We were the first to assess the changes in serum survivin during treatment and show that increased survivin levels after chemotherapy were a marker of a better treatment outcome in MM, emphasizing the importance of survivin measurement before and after chemotherapy. Our sample size was comparable to other studies or even bigger, especially taking into account the rarity of MM. In the survival analysis, we were able to detect with 80% power differences in HR of approximately 0.5 or lower. However, our results need to be confirmed in an independent patient cohort.

Even though the role of survivin in cancer treatment is complex, our results suggest that measurement of serum survivin levels before and during chemotherapy could serve as a noninvasive biomarker predicting a response to treatment in MM, potentially contributing to a better treatment outcome in these patients.

## Figures and Tables

**Table 1:** Patients' characteristics.

Characteristic	All patients (*N* = 78) *N* (%)	Exposed to asbestos (*N* = 63) *N* (%)
Gender		
Male	66 (84.6)	54 (85.7)
Female	12 (15.4)	9 (14.3)
Age		
Years, median (25–75%)	64.0 (57.8–70.3)	65.0 (58.0–70.0)
Asbestos exposure		
Exposed	63 (80.8)	63 (100.0)
Unexposed	15 (19.2)	
Smoking		
Nonsmokers	36 (46.2)	30 (47.6)
Smokers	42 (53.8)	33 (52.4)
Stage		
I	6 (7.7)	6 (9.5)
II	21 (26.9)	17 (27.0)
III	26 (33.3)	19 (30.2)
IV	18 (23.1)	17 (27.0)
Peritoneal	7 (9.0)	4 (6.3)
Histological type		
Epitheloid	60 (76.9)	48 (76.2)
Biphasic	11 (14.1)	10 (15.9)
Sarcomatoid	4 (5.1)	3 (4.8)
Not characterized	3 (3.8)	2 (3.2)
Eastern Cooperative Oncology Group performance status		
0	33 (42.3)	28 (44.4)
1	26 (33.3)	21 (33.3)
2	19 (24.4)	14 (22.2)
CRP		
mg/L, median (25–75%)	21 (6–62)	22 (6–58)
First-line chemotherapy		
Gemcitabine and cisplatin	53 (67.9)	43 (68.3)
Pemetrexed and cisplatin	25 (32.1)	20 (31.7)
Response rate^*∗*^		
CR or PR	29 (37.7)	23 (37.1)
SD or progress	48 (62.3)	39 (62.9)
Overall survival		
Months, median (25–75%)	20.0 (11.6–47.8)	18.0 (11.6–28.7)
Progression-free survival		
Months, median (25–75%)	7.9 (5.6–13.9)	7.8 (5.6–13.1)

^*∗*^Data missing for 1 patient; CR: complete response; *N*: number of patients; PR: partial response; SD: stable disease.

**Table 2:** Survivin levels before chemotherapy and change in survivin levels after chemotherapy and MM treatment outcome.

Treatment outcome	Survivin levels before chemotherapy (pg/mL, median (25% to 75%))		Change in survivin levels (pg/mL, median (25% to 75%))	
	All patients (*N* = 77)	Patients exposed to asbestos (*N* = 62)	All patients (*N* = 77)	Patients exposed to asbestos (*N* = 62)
Complete response	0 (0–37.0)	0 (0–37.0)	114.3 (6.0 to 304.5)	114.3 (6.0 to 304.5)
Partial response	33.9 (0–803.4)	4.1 (0–174.8)	61.6 (0 to 163.9)	62.2 (0 to 110.3)
Stable disease	0 (0–124.9)	0 (0–71.9)	0 (−2.0 to 66.9)	0 (−4.1 to 55.3)
Progress	199.7 (38.0–3102.7)	166.9 (62.0–2377.0)	−45.2 (−975.4 to 0)	−88.6 (−1370.9 to −39.8)
Kruskal-Wallis test statistic	7.951	6.561	8.892	14.365
*p* value	0.047	0.087	0.031	0.002

*N*: number of patients.

**Table 3:** Change in survivin levels after chemotherapy and response rate.

Observed response	All patients (*N* = 77)		Patients exposed to asbestos (*N* = 62)	
	Decreased survivin levels *N* (%)	Increased survivin levels *N* (%)	Decreased survivin levels *N* (%)	Increased survivin levels *N* (%)
CR + PR	9 (20.9)	20 (58.8)	6 (17.1)	17 (63.0)
SD + PD	34 (79.1)	14 (41.2)	29 (82.9)	10 (37.0)

CR: complete response; *N*: number of patients; PR: partial response; SD: stable disease.
